# Supplementary material for: In Leishmania major, the Homolog of the Oncogene PES1 May Play a Critical Role in Parasite Infectivity
Source: Int J Mol Sci. 2021 Nov 22;22(22):12592. doi: 10.3390/ijms222212592 (PMC8618447; doi:10.3390/ijms222212592)
Supplement: Supplementary file 1 [file ijms-22-12592-s001.zip › ijms-1424049-supplementary.pdf]

```
L. braziliensis   MAHKKQAWAKRVKKERFKKKYLTRMQATRLLQMESIQFRRLCILKGVYPRALTRSKQKQSGNEKQYYLAREIKWLVRDHIAEKMMTYRAWEKKVKRAEAMGRADDLKALQSSRVKPRHSL 120
L. mexicana       MAHKKQAWAKRVKKERFKKKFLTRMQATRLLQMESVQFRRLCILKGIYPRALQRSKQKQSGNEKQYYLAREIKWLVRDQIAEKMMAYRAWEKKVKRAEAMGRAEDLKVLQSSRVKPRHSL 120
L. major          MVHKKQAWAKRVKKERFKKKFLTRMQATRLLQMESVQFRRLCILKGIYPRALNRSKQKQSGNEKQYYLAREIKWLVRDQIAEKMMAYRAWEKKVKRAEAMGRSEDLKVLQTSRVKPRHSL 120
L. infantum       MAHKKQAWAKRVKKERFKKKFLTRMQATRLLQMESVQFRRLCILKGIYPRALNRSKQKQSGHEKQYYLAREIKWLVRDQIAEKMMAYRAWEKKVKRAEAMGRSEDLKVLQSSRVKPRHSL 120
L. donovani       MAHKKQAWAKRVKKERFKKKFLTRMQATRLLQMESVQFRRLCILKGIYPRALNRSKQKQSGHEKQYYLAREIKWLVRDQIAEKMMAYRAWEKKVKRAEAMGRSEDLKVLQSSRVKPRHSL 120
T. cruzi          –MHRKQVWAKRVKKERLKKKFLTRMQATRLLQVDSVHFRRLCILKGIYPRALARSKQKQSGNDKQYYLAREIKWLVRDHVAERMMAYRAWEKKVRRAEAMGLADELKVLRSKKAKPQYSL 119
T. brucei brucei –MHRKQAWSKRVKKERQKKKYLTRMQATRLLQVDSIHFRRLCILKGIYPRALNRSKQKQSGNDKQYYLAREIKWLVRDHLADRMMAFRAWEKKVRRAEAMRLTEDLKVLHSNKVKPKYSL 119
                    *:**.*:******* ***:***********::*::*********:***** ********::**************::*::**::*******:*****  :::**.*::.:.**::**

L. braziliensis   VATIKERYPYFIDAIRDVDDAMSMIHMYAFLSPEIKSESTIEIHHSLTSGLSEKAKEMCHRWNRYIARAHVLTKGFISIKGYYYEAIIKGERVRWLCPHEYAHRFPPGIQQYVMLSFLEF 240
L. mexicana       VATIKERYPYFIDAIRDVDDAMSMIHLYAFLSPEIKSDTTIEIHHSLTSGMSEKAKEMCERWDRYVARARVLTKGFISIKGYYYEAIVKGERVRWLCPHEYAHRFPPGIQQYVMLSFLEF 240
L. major          VATIKERYPYFIDAIRDVDDAMSMIHLYAFLSPEIKSDTTIEIHHSLTSGLSERAKEVCERWDRYVARARVLTKGFISIKGYYYEAIVKGERVRWLCPHEYAHRFPPGIQQYVMLSFLEF 240
L. infantum       VATIKERYPYFIDAIRDVDDAMSMIHLYAFLSPEIKSDTTIEIHHSLTSGMSEKAKEVCERWDRYVARARVLTKGFISIKGYYYEAIVKGERVRWLCPHEYAHRFPPGIQQYVMLSFLEF 240
L. donovani       VATIKERYPYFIDAIRDVDDAMSMIHLYAFLSPEIKSDTTIEIHHSLTSGMSEKAKEVCERWDRYVARARVLTKGFISIKGYYYEAIVKGERVRWLCPHEYAHRFPPGIQQYVMLSFLEF 240
T. cruzi          VATIKERYPFFIDAVRDIDDAMSMIALYAFLSPEVMSETTIETHHALTSGLHERARGIIARWNRYVARAQVLSKGFISIKGYYFEAIVRGERVRWLCPHEYAHKFPPGIQQYITLSFLEF 239
T. brucei brucei VATIKERYPYFADAVRDIDDAMSMIALYAFLSPEVASESTIETHHTLTSGLHRRARETCERWNSYVERAQALSKGFISIKGYYFEAIVRGERVRWLCPHEYAHKFPAGVQQYIMISFLEF 239
                 *********:* **:**:******* :*******: *::*** **:****: .:*:    **: *: **:.*:**********:***::**************:** *:***: :*****

L. braziliensis   YLEMMKFVLFKLESDLARDEADRLAIEDEEGLTRANAEDFASGAALAVLDVGANQAQAK-VKEAESKRSLMEEELLKVRELFRGLTFYISREVPAKHFALIINACGGRVATDYVASNITH 359
L. mexicana       YLEMMKFVLFKLESDLARDEADRLAAEDEEGVARANAEDFANGAALAVLDVGANQAQAKEVKEAESKRSLMEEELQKVRELFRGLTFFISREVPAKHFALVIKACGGRVATDYVPSNITH 360
L. major          YLEMMKFVLFKLESDLARDEADRLAAEDEEGVTRANAEDFANGAALAVLDVGANQAQAKAVKEAESKRSLMEEELHKVRELFRGLTFFISREVPAKHFALVINACGGRVATDYVPSNITH 360
L. infantum       YLEMMKFVLFKLESDLARDEADRLAAEDEEGVTRANAEDFANGGALAVLDVGANGAQAKEVKEAESKRSLMGEELHKVRELFRGLTFFISREVPSKRFALVINACGGRVATDYVPSNITH 360
L. donovani       YLEMMKFVLFKLESDLARDEADRLAAEDEEGVTRANAEDFANGGALAVLDVGANGAQAKEVKEAESKRSLMGEELHKVRELFRGLTFFISREVPSKRFALVINACGGRVATDYVPSNITH 360
T. cruzi          YVELMQFVLYKLEHELQRDVAEQQEAEDEENNTH--AEDFRLPDKEMT------QSISDVKKDTLKKGELMQEELRKARRVFDGLTFYISREVPHKHATLIIEACGGRVATAYAADNITH 351
T. brucei brucei YLELMRFVLFKLER----DLAEKQKAEEEEPNLN--AENFDTTNKETV------QGLGGTVGQVAKRVELMDEELRKVRRVFEGLSFYISREVPHKHAALVIKACGGRVATGYVAENITH 347
                 *:*:*:***:***     * *::   *:**   .  **:*        .        . .    :. .: .** *** *.*.:* **:*:****** *: :*:*:******** *. .****

L. braziliensis   VVVDRPALPPGWQQHDQMEYVQPQYIFDCLNARQMLPVTGYRIGEDLPPHVSPFSVSITNSAEDNAAVEQVKKDHPRIVGYVPARVHEIRKLINPSYSPVDPEGKVAQLEDEYSDEEMHV 479
L. mexicana       VVVDRPSLPPGMKRYDPLEYVQPQYIFDCLNARLMLPVTGYRMGEELPPHVSPFSVSITNSAEDNAAVEQVKKDHPKIVGYVPARVHEVRKLINPSYSAVDPEGKVAHLEDEYSDEECHV 480
L. major          VVVDRPALPPGMKKHDQLEYVQPQYIFDCLNARLVLPVTGYRIGEELPPHVSPFSVSITNSAEDNAAVVQVKKDHPRIVGYVPARVHEIRKLINPSYSAVDPEGKVAHLEDEYSDEESHV 480
L. infantum       VVVDRPALPPGMKKHDQLEYVQPQYIFDCLNARLMLPVTGYRIGEELPPHVSPFSVSITNSAEDNAAVEQVKKDHPRIVGYVPARVHEIRKLINPSYSAVDPEGKVAHLEGEYSDEESHV 480
L. donovani       VVVDRPALPPGMKKHDQLEYVQPQYIFDCLNARLMLPVTGYRIGEELPPHVSPFSVSITNSAEDNAAVEQVKKDHPRIVGYVPARVHEIRKLINPSYSAVDPEGKVAHLEGEYSDEESHV 480
T. cruzi          FVVDRPALPPGFERVDRIEYVQPQYLFDCLNARMLLPPNGYRIGEELPPHVSPFTVAITNSPEDIAAVEEAKKNHPKIVSYVPKRVHEIRALVNPQYTPLDPEGKASDLLLHDESDEDHA 471
T. brucei brucei FVVDRPALPPGFEKDDRIEYVQPQYLFDCLNARLLLPPNGYRIGEELPPHVSPFTVAITNSTEDIAAVEEARRDHPKIVSYVPKRVHEIRKLINPQYTPTDPDGKAAELLLEEESDEENV 461
                 .*****:**** :: * :*******:******* :** .***:**:********:*:*** *** *** :.:::**:**.*** ****:* *:**.*:  **:**.:.*  . ..:* :.

L. braziliensis   AVPEMDMEDDVSLSGDELAEARKKPGWQEEEVTEEVQRPKLSAFKVKKQREMNLMNAPTNEVVARRRQALRKAQENLRQSETPEARLQRKMSEVKRQEAVTRKMQLQVARKKAARFYKMV 599
L. mexicana       AVPEMDMEDDVSLSGDELAEARRKPAWQEEEVTEEVQRPKLSAFKVKKQREMSLMNAPTNEIVARRRQALRKSQEKSRQAETSEARLQRRMSEVKRQEAATRKMQLQVARKKAARFYKMV 600
L. major          AVPEMDMEDDVSLSGDELAEARRKPAWQEEEVTEEVQRPKLSAFKVKKQREMNLMNAPTNEVVARRRQALRKAQEKSRQTETSEARLQRKMSEVKRQEAATRKMQLQVARKKAARFYKMV 600
L. infantum       AVPEMDMEDDVSLSGDELAEARRKPAWQEEEVTEEVQRPKLSAFKVKKQREMNLMNAPTNEVVARRRQALRKAQEKSRQTETSEARLQRKMSEVKRQEAATRKMQLQVARKKAARFYKMI 600
L. donovani       AVPEMDMEDDVSLSGDELAEARRKPAWQEEEVTEEVQRPKLSAFKVKKQREMNLMNAPTNEVVARRRQALRKAQEKSRQTETSEARLQRKMSEVKRQEAATRKMQLQVARKKAARFYKMI 600
T. cruzi          LVPELGADDDVSLSGDELLEASHRPKWEEEEVTENVQRSKLSALKVAKQRKMNLMNAPTDEVVARRRLAQLKSRESRQQQEPSGQRLKRKLAEMKRQEAATRKMQLQVARKKAARYYKMV 591
T. brucei brucei AAQELDRDDDVQLSGDELQEARRRPKWEDEQVTESVERTKLSALKVRQQRELNLMNAPTNEAVARRRLAQQKAKEARKQQETDEHRLRRKLADVKRQEVATRKMQLQVARKKAARYYKMV 587
                 . *:. :***.****** ** ::* *::*:***.*:* ****:** :**::.******: * ***** *  *::*  :* *    **:*::::****..**************:***:

L. braziliensis   SGVVQGTAKKAATLEAKAKHIAEGKLHKTEDGKGLVNARLAARRQRAEAKGKKLKEKKADNPYKKLPKWVQ 670
L. mexicana       SGVVQGTAKKAATLEAKAKHIAEGKLHKTEDGKGLVNTRLEARRQRAEAKGKKLKEKKADNPYKKLPKWVQ 671
L. major          SGVVQGTAKKVATLEAKAKHIAEGKLRKTEDGKGLVNKRLEARRQRAEAKGKKLKERKAGNPYKKLPKWVQ 671
L. infantum       SGVVQGTAKKAATLEAKAKHIAEGKLHKTEDGKGLVNTRLEARRQRAEAKSKKLKERKAGNPYKKLPKWVQ 671
L. donovani       SGVVQGTAKKAATLEAKAKHIAEGKLHKTEDGKGLVNTRLEARRQRAEAKSKKLKERKAGNPYKKLPKWVQ 671
T. cruzi          NSVVQGSQRREKMLEAKAKQVADGRIAKKEDGRELVNVRVEAKRARAAASGKPLREKKAANPYKKLPKWVR 662
T. brucei brucei NGVVQGNERREKMLTAKAKQLSEGRISAAKDGSGLVNNKLEARRAKAEAAGKKLKEKKPSNPYSKLPKWVR 658
                 ..****. ::   * ****::::*::   :**  *** :: *:* :* * .* *:*:* ***.******:
```

S1

```
LmjF.04.0810       ATGGTCCATAAGAAGCAGGCATGGGCGAAGCGGGTGAAGAAGGAGCGCTTCAAGAAGAAATTTCTGACGCGCATGCAGGCGACGCGGCTACTGCAGATGGAGTCCGTTCAGTTCCGCCGC 120
LmjPES_sequenced   ATGGTCCATAAGAAGCAGGCATGGGCGAAGCGGGTGAAGAAGGAGCGCTTCAAGAAGAAATTTCTGACGCGCATGCAGGCGACGCGGCTACTGCAGATGGAGTCCGTTCAGTTCCGCCGC 120
                   ************************************************************************************************************************

LmjF.04.0810       CTCTGCATCCTCAAGGGCATCTACCCCCGCGCCCTCAACCGCAGCAAGCAGAAACAAAGTGGGAACGAGAAGCAGTACTATCTCGCCCGTGAGATCAAGTGGCTGGTGCGCGACCAGATA 240
LmjPES_sequenced   CTCTGCATCCTCAAGGGCATCTACCCCCGCGCCCTCAACCGCAGCAAGCAGAAACAAGGTGGGAACGAGAAGCAGTACTATCTCGCCCGTGAGATCAAGTGGCTGGTGCGCGACCAGATA 240
                   ********************************************************* **************************************************************

LmjF.04.0810       GCGGAGAAGATGATGGCATATCGGGCATGGGAGAAGAAGGTGAAGCGGGCAGAGGCGATGGGCCGCTCCGAGGACCTCAAGGTGCTGCAGACCTCACGCGTGAAGCCGCGCCACAGCCTG 360
LmjPES_sequenced   GCGGAGAAGATGATGGCATATCGGGCATGGGAGAAGAAGGTGAAGCGGGCAGAGGCGATGGGCCGCTCCGAGGACCTCAAGGTGCTGCAGACCTCACGCGTGAAGCCGCGCCACAGCCTG 360
                   ************************************************************************************************************************

LmjF.04.0810       GTGGCGACGATCAAGGAGCGCTACCCATACTTCATCGACGCCATCCGTGACGTGGATGACGCCATGTCGATGATCCACCTTTACGCCTTCCTTAGCCCGGAGATCAAGTCCGATACCACC 480
LmjPES_sequenced   GTGGCGACGATCAAGGAGCGCTACCCATACTTCATCGACGCCATCCGTGACGTGGATGACGCCATGTCGATGATCCACCTTTACGCCTTCCTTAGCCCGGAGATCAAGTCCGATACCACC 480
                   ************************************************************************************************************************

LmjF.04.0810       ATCGAGATACATCACTCCCTCACCTCCGGCCTGAGCGAGAGGGCCAAGGAGGTGTGCGAACGCTGGGACCGCTACGTCGCACGCGCGCGTGTACTGACGAAGGGCTTCATCTCCATCAAG 600
LmjPES_sequenced   ATCGAGATACATCACTCCCTCACCTCCGGCCTGAGCGAGAGGGCCAAGGAGGTGTGCGAACGCTGGGACCACTACGTCGCACGCGCGCGTGTACTGACGAAGGGCTTCATCTCCATCAAG 600
                   ********************************************************************** *************************************************

LmjF.04.0810       GGCTACTACTACGAGGCGATTGTCAAGGGCGAGCGGGTGCGGTGGCTGTGCCCGCACGAGTACGCTCACCGCTTCCCCCCGGGCATCCAGCAATACGTGATGCTCAGCTTCCTCGAATTC 720
LmjPES_sequenced   GGCTACTACTACGAGGCGATTGTCAAGGGCGAGCGGGTGCGGTGGCTGTGCCCGCACGAGTACGCTCACCGCTTCCCCCCGGGCATCCAGCAATACGTGATGCTCAGCTTCCTCGAATTC 720
                   ************************************************************************************************************************

LmjF.04.0810       TACCTGGAGATGATGAAGTTCGTCCTGTTCAAGCTCGAGTCGGACCTCGCGCGCGATGAGGCGGACCGCCTGGCGGCCGAGGATGAGGAAGGTGTGACCCGCGCGAACGCCGAGGACTTC 840
LmjPES_sequenced   TACCTGGAGATGATGAAGTTCGTCCTGTTCAAGCTCGAGTCGGACCTCGCGCGCGATGAGGCGGACCGCCTGGCGGCCGAGGATGAGGAAGGTGTGACCCGCGCGAACGCCGAGGACTTC 840
                   ************************************************************************************************************************

LmjF.04.0810       GCGAACGGTGCGGCGCTGGCCGTGCTGGATGTAGGCGCGAACCAGGCGCAGGCCAAGGCGGTGAAGGAGGCGGAGTCGAAGCGCTCTTTGATGGAGGAAGAGCTGCACAAGGTGCGCGAG 960
LmjPES_sequenced   GCGAACGGTGCGGCGCTGGCCGTGCTGGATGTAGGCGCGAACCAGGCGCAGGCCAAGGCGGTGAAGGAGGCGGAGTCGAAGCGCTCTTTGATGGAGGAAGAGCTGCACAAGGTGCGCGAG 960
                   ************************************************************************************************************************

LmjF.04.0810       CTGTTCCGCGGGCTAACCTTCTTCATATCGCGTGAGGTGCCCGCGAAGCACTTCGCCCTCGTCATCAACGCCTGCGGCGGCCGTGTGGCGACCGACTACGTGCCGTCGAACATCACTCAC 1080
LmjPES_sequenced   CTGTTCCGCGGGCTAACCTTCTTCATATCGCGTGAGGTGCCCGCGAAGCACTTCGCCCTCGTCATCAACGCCTGCGGCGGCCGTGTGGCGACCGACTACGTGCCGTCGAACATCACTCAC 1080
                   ************************************************************************************************************************

LmjF.04.0810       GTCGTTGTTGACCGCCCCGCGCTGCCGCCAGGGATGAAAAAGCATGACCAGCTGGAGTACGTGCAGCCGCAGTACATCTTCGACTGCCTGAACGCGCGGCTGGTGCTCCCGGTGACGGGC 1200
LmjPES_sequenced   GTCGTTGTTGACCGCCCCGCGCTGCCGCCAGGGATGAAAAAGCATGACCAGCTGGAGTACGTGCAGCCGCAGTACATCTTCGACTGCCTGAACGCGCGGCTGGTGCTCCCGGTGACGGGC 1200
                   ************************************************************************************************************************

LmjF.04.0810       TACCGCATTGGTGAGGAGCTGCCGCCGCACGTGTCGCCCTTCTCTGTCTCCATCACGAACTCCGCCGAGGACAACGCGGCGGTGGTGCAGGTGAAGAAGGACCACCCGAGGATTGTGGGC 1320
LmjPES_sequenced   TACCGCATTGGTGAGGAGCTGCCGCCGCACGTGTCGCCCTTCTCTGTCTCCATCACGAACTCCGCCGAGGACAACGCGGCGGTGGTGCAGGTGAAGAAGGACCACCCGAGGATTGTGGGC 1320
                   ************************************************************************************************************************

LmjF.04.0810       TACGTGCCGGCGCGCGTGCATGAGATCCGCAAGCTGATCAACCCCTCTTACTCTGCGGTCGACCCAGAGGGGAAGGTGGCGCATCTGGAGGACGAGTACAGCGACGAAGAGTCTCACGTG 1440
LmjPES_sequenced   TACGTGCCGGCGCGCGTGCATGAGATCCGCAAGCTGATCAACCCCTCTTACTCTGCGGTCGACCCAGAGGGGAAGGTGGCGCATCTGGAGGACGAGTACAGCGACGAAGAGTCTCACGTG 1440
                   ************************************************************************************************************************

LmjF.04.0810       GCGGTGCCGGAGATGGACATGGAGGACGATGTCTCGCTCTCCGGTGACGAGCTTGCGGAGGCACGCAGGAAGCCGGCGTGGCAGGAAGAGGAGGTCACGGAGGAGGTGCAGCGGCCGAAG 1560
LmjPES_sequenced   GCGGTGCCGGAGATGGACATGGAGGACGATGTCTCGCTCTCCGGTGACGAGCTTGCGGAGGCACGCAGGAAGCCGGCGTGGCAGGAAGAGGAGGTCACGGAGGAGGTGCAGCGGCCGAAG 1560
                   ************************************************************************************************************************

LmjF.04.0810       CTGTCGGCGTTCAAGGTGAAGAAGCAGCGCGAGATGAACCTGATGAACGCGCCGACGAACGAAGTGGTGGCGCGGCGGCGGCAGGCACTGCGCAAGGCGCAGGAGAAGTCACGTCAGACG 1680
LmjPES_sequenced   CTGTCGGCGTTCAAGGTGAAGAAGCAGCGCGAGATGAACCTGATGAACGCGCCGACGAACGAAGTGGTGGCGCGGCGGCGGCAGGCACTGCGCAAGGCGCAGGAGAAGTCACGTCAGACG 1680
                   ************************************************************************************************************************
```

S2 **(1/2)**

```
LmjF.04.0810         GAGACGTCCGAGGCACGTCTGCAGCGCAAGATGAGCGAGGTGAAGCGCCAGGAGGCGGCGACGCGCAAGATGCAGCTGCAGGTCGCCCGCAAGAAGGCAGCACGTTTTTACAAGATGGTC 1800
LmjPES_sequenced     GAGACGTCCGAGGCACGTCTGCAGCGCAAGATGAGCGAGGTGAAGCGCCAGGAGGCGGCGACGCGCAAGATGCAGCTGCAGGTCGCCCGCAAGAAGGCAGCACGTTTTTACAAGATGGTC 1800
                     ************************************************************************************************************************

LmjF.04.0810         AGCGGTGTGGTGCAGGGCACGGCGAAGAAGGTGGCGACGCTGGAGGCAAAGGCAAAGCACATCGCCGAAGGGAAGCTGCGTAAGACCGAGGATGGCAAGGGACTTGTCAACAAGCGGCTG 1920
LmjPES_sequenced     AGCGGTGTGGTGCAGGGCACGGCGAAGAAGGTGGCGACGCTGGAGGCAAAGGCAAAGCACATCGCCGAAGGGAAGCTGCGTAAGACCGAGGATGGCAAGGGACTTGTCAACAAGCGGCTG 1920
                     ************************************************************************************************************************

LmjF.04.0810         ATCGCCGAAGGGAAGCTGCGTAAGACCGAGGATGGCAAGGGACTTGTCAACAAGCGGCTGGAGGCGCGACGGCAGCGAGCTGAGGCGAAGGGCAAGAAGCTCAAGGAGAGGAAGGCTGGC 1980
LmjPES_sequenced     ATCGCCGAAGGGAAGCTGCGTAAGACCGAGGATGGCAAGGGACTTGTCAACAAGCGGCTGGAGGCGCGACGGCAGCGAGCTGAGGCGAAGGGCAAGAAGCTCAAGGAGAGGAAGGCTGGC 1980
                     ************************************************************************************************************************

LmjF.04.0810         AACCCGTACAAGAAACTGCCCAAGTGGGTGCAGTAA 2016
LmjPES_sequenced     AACCCGTACAAGAAACTGCCCAAGTGGGTGCAGTAA 2016
                     ************************************
```

S2 (2/2)
